# Rapid preparation of *Candida* genomic DNA: combined use of enzymatic digestion and thermal disruption

**DOI:** 10.1186/s13568-022-01500-z

**Published:** 2023-01-02

**Authors:** Zhengxin He, Xiaosai Huo, Jingzi Piao

**Affiliations:** 1 Basic Medical Laboratory, The 980th Hospital of PLA Joint Logistical Support Force (Bethune International Peace Hospital), 398 Zhongshan Road, Shijiazhuang, 050082 Hebei People’s Republic of China; 2 College of Plant Protection, Shenyang Agricultural University, 120 Dongling Road, Shenyang, 110866 Liaoning People’s Republic of China

**Keywords:** *Candida*, DNA preparation method, PCR, Clinical laboratory

## Abstract

Nucleic acid based molecular technologies are the most promising tools for the early diagnosis of *Candida* infection. A simple and effective DNA preparation method is of critical for standardizing and applying molecular diagnostics in clinic laboratories. The goal of this study was to develop a *Candida* DNA preparation method that was quick to do, easy to perform, and bio-safe. Snailase and lyticase were screened and combined in this work to enhance the lysis of *Candida* cells. The lysis solution composition and metal bath were optimized to boost amplification efficiency and biosafety. A duplex real-time PCR was established to evaluate the sensitivity and specificity of the preparation method. Using the supernatant from the rapid preparation method as templates, the duplex PCR sensitivities for five common *Candida* species were determined to be as low as 10^0^ CFUs. When compared to conventional preparation methods, the samples prepared by our method showed higher PCR detection sensitivity. PCR identification and ITS sequencing were 100% consistent, which was better than biochemical identification. This study demonstrates a rapid method for *Candida* DNA preparation that has the potential to be used in clinical laboratories. Meanwhile, the practical application of the method for clinical samples needs to be proven in future investigations.

**Supplementary Information:**

The online version contains supplementary material available at 10.1186/s13568-022-01500-z.

## Introduction

Invasive candidiasis (IC), including candidemia, is a significant cause of morbidity and death, particularly in individuals with severe medical conditions (Cleveland et al. 2012; Lamoth et al. 2018; Magill et al. 2014). Even when IC patients were given antifungal medication, the death rate in severely sick patients was as high as 58.6% (Al-Dorzi et al. 2020). Previous epidemiological data showed that *Candida* species are among the top four pathogens responsible for nosocomial bloodstream infection (Pfaller and Diekema 2007; Wisplinghoff et al. 2004). The major 5 leading *Candida* pathogens that cause IC were: *Candida albicans*, *Candida glabrata*, *Candida tropicalis*, *Candida parapsilosis* and *Candida krusei*. Although *C. albicans* is the most common cause of *Candida* infection, the prevalence of IC caused by non-*albicans* spp. has steadily grown (Borjian Boroujeni et al. 2021). Notably, the human race has always been vulnerable to emerging yeast infection. *Candida auris*, a novel pathogen known as a "superbug fungus," is a multidrug-resistant yeast that has caused invasive infection and mortality in recent years (Chakrabarti and Singh 2020; Plachouras et al. 2020; Satoh et al. 2009). In 2019, many countries, including the United States, experienced nosocomial *C. auris* outbreaks. According to Adams E et al*.*, approximately half of *C. auris* infected individuals died within 90 days of being identified with the fungus (Adams et al. 2018).

Traditional laboratory diagnosis and empirical therapy strategies revealed significant limits in dealing with IC, suggesting that prompt identification and susceptibility testing of *Candida* species may not always be possible. Culture-based identification and characterization approaches are sometimes hampered by long turnaround times and poor sensitivities (Clancy and Nguyen 2013; Ness et al. 1989; Pfeiffer et al. 2011). Different guidelines have recently suggested the G test, which is designed to detect (1,3)-D-glucan, for IC screening(Hage et al. 2019; Martin-Loeches et al. 2019). However, this approach does not give enough information for IC management, such as *Candida* species and yeast susceptibility test results. Because glucans are a non-specific polysaccharide component of fungal cell walls that may be detected in a wide range of species, false positive results are a methodological problem for the G test(Egger et al. 2018; Ito et al. 2018).

Technological advances in nucleic acid-based molecular diagnostics bring new insights into IC diagnosis. Several nucleic acid detection tests, such as polymerase chain reaction (PCR), hybridization, and the loop-mediated isothermal amplification (LAMP), have been shown to identify the infection even in its early stages (Inácio et al. 2008). Among them, PCR was regarded as the most representative and promising diagnostic tool because to its high sensitivity, specificity, reproducibility, and short detection time (Derveaux et al. 2010; Huggett et al. 2005). The majority of nucleic acid-based molecular diagnostic methods involve two steps: DNA extraction and amplification. The fundamental assurance for amplification is the quality and efficiency of DNA preparation. Tough cell walls of *Candida* spp., which are made up of Chitin, glucan, mannan, and glycoprotein, make DNA preparations challenging.

To address this issue, the researchers followed different strategies (Liu et al. 2000; Romanelli et al. 2014). However, most of these methods are incapable of meeting the criteria of clinical laboratories for easy standardization and biosafety. Thus improve the DNA preparation method would boost the application of IC molecular diagnostics. Here we aimed to demonstrate a *Candida* DNA preparation method with shorter sample turnaround time, easy to perform, and bio-safe. Furthermore, the prepared DNA samples can be directly applied to the subsequent PCR reaction, removing the need for traditional procedures of DNA extraction and purification.

## Materials and methods

### Candida strains and culture

*C. albicans* strain SC5314 was used in this study (He et al. 2015). The standard *Candida* species involved, including *C. tropicalis* ATCC1369, *C. glabrata* ATCC15126, *C. parapsilosis* ATCC22019 and *C. krusei* ATCC6258, as well as a standard *Saccharomyces cerevisiae* strain ATCC9763 were obtained from the American Type Culture Collection. The fungi were cultured to exponential phase at 35 °C, 5% CO_2_ in yeast extract peptone dextrose (YPD) medium (1% yeast extract, 2% peptone, 2%D-glucose) and harvested by centrifugation at 400 × g for 5 min. After two washes with PBS, the fungal density was determined using a cell counting plate. A *Candida* count of 5 × 10^7^ CFUs was used to choose the suitable digestive enzymes; a *Candida* count of 5 × 10^5^ CFUs was utilized to validate the ideal lysis conditions, which may be closer to the fungal load in real applications.

### Candida lysis ability of digestive enzymes

Digestive enzymes, including snailase (Takara Biotechnology, China), lyitcase (Sigma, USA), zymolyase (Zymo Research, USA) and glucanase (Sigma, USA), were tested for their capacity to lyse *Candida* cells. Aliquots of enzyme at different concentrations were prepared according to the manufacturer’s instructions.

The precipitated *Candida* cells were resuspended in 1 mL of suspension buffer (0.5 M sorbitol, 25 mM EDTA, pH = 8.0) containing 2 μL of β-mercaptoethanol. The different digestive enzymes then were added to each Eppendorf tube at the following concentrations: (1) snailase: 0, 130, 650, 1040 and 2080 μg/mL; (2) lyitcase: 0, 100, 200, 500 and 800 U/mL; (3) zymolyase: 0, 40, 80, 160 and 320 U/mL; and (4) glucanase: 0, 40, 80, 160 and 320 U/mL. All of the digestive reaction systems described above were incubated at the temperatures recommended by the enzymes, which were 35, 30, 30 and 50 °C for snailase, lyitcase, zymolyase and glucanase, respectively. The lysing effects were assessed after 5 h by counting the remaining intact *Candida* blastospores with a cell counting plate.

### Preparation of genomic DNA control

Overnight cultured *C. albicans* SC5314 cells were collected by centrifugation. Following a PBS wash, the cells were treated with a yeast lytic enzyme kit (Solarbio Life Sciences, China). SC5314 cells were resuspended in 470 µL of sorbitol buffer (0.6 M sorbitol, 5 mM EDTA, 50 mM Tris pH 7.4) and incubated for 10 min at room temperature. Then, 25 µL of enzyme solution and 5 µL of β-mercaptoethanol were added into the SC5314 suspension. The mixture was incubated in a water bath at 30 °C for 4 h before being centrifuged at 14,000 g for 30 min. The genomic DNA was extracted from the collected precipitates using the Yeast DNA Extraction Kit (Omega BioTec, USA) according to manufacturer’s instructions.

### Analyze PCR inhibitors in the lysis solution

WE designed an interference experiment to evaluate the impact of the components on the PCR because the lysis solution comprises a variety of components. The purified SC5314 genomic DNA was used as the PCR template. Snailase, lyticase, β-mercaptoethanol, sorbitol and EDTA were separately added into the 50 µL PCR reaction systems to observe their effects on amplification. Primer and probe sequences were as follows: forward primer, 5′- GGG TTT GCT TGA AAG ACG GTA -3′; reverse primer, 5′- TTG AAG ATA TAC GTG GTG GAC GTT A -3′; TaqMan probe, 5′-FAM- ACC TAA GCC ATT GTC AAA GCG ATC CCG -TAMRA-3′. PCR reactions were performed in 50 µl volumes, containing 2 µl of each primer (forward and reverse) at a concentration of 10 µmol/L, 25 µl of 2 × Taq PCR Mix (Tiangen Biotech, China), 2 µl Taqman probe, 4 µl of DNA template (∼50 ng) and a specified volume of lysate components; The reaction volume was adjusted to 50 μL with double-distilled water. The qPCR was performed using a SLAN 96P real time PCR System (HONGSHI, China), and cycling conditions were as follows: initial denaturation at 95 °C for 2 min, followed by 40 cycles of 94 °C for 15 s and 60 °C for 1 min.

### Impact of high-temperature metal bath

Cultured *C. albicans* SC5314 cells were washed with PBS and divided into two equal aliquots. Following centrifugation, the aliquots were incubated with optimized lysis solution at 30 °C for 1 h. Then, one aliquot was heated at 100 °C in a DB100C metal bath (JOANLAB, China) for 10 min, while the second aliquot was left untreated. After centrifuging the lysates for 5 min, the supernatants were utilized. The parameters of the amplification curve and cycle threshold (Ct) values were used to assess the effect of high temperature on PCR performance.

### Duplex real-time PCR

Prior to developing the multiplex real-time PCR test, singleplex real-time PCR techniques were optimized to identify the five *Candida* species. The primers and probes were designed as previously reported (Brinkman et al. 2003; Guiver et al. 2001; Guo et al. 2016). The matrix dilution method was used to establish the optimal concentration of primers and probes.

As shown in Table 1, five sets of specialized primers/probes and one set of universal primers/probes were matched into three duplex PCR reactions. The 50-μl PCR reaction system, equipment, and amplification conditions were configured exactly as described in “Analyze PCR inhibitors in the lysis solution.”

**Table 1:** *Candida* TaqMan primer and probe sequences

Species	Sequences (5’to 3’)^a^	Target^b^
Duplex 1		
* Candida albicans*	F: GGG TTT GCT TGA AAG ACG GTA R: TTG AAG ATA TAC GTG GTG GAC GTT A P: VIC-ACC TAA GCC ATT GTC AAA GCG ATC CCG-TAMRA	ITS2
Universal *Candida spp*.	F:GGA TCT CTT GGT TCT CGC ATC R:AAC GAC GCT CAA ACA GGC AT P:FAM-CGC AAT GTG CGT TCA A-TAMRA	ITS2
Duplex 2		
* Candida glabrata*	F:TTT CTC CTG CCT GCG CTT AA R:ACG CAC ACT CCC AGG TCT TT P:VIC-AGA ACA CCC ACC AAC CGC GCA-TAMRA	ITS2
* Candida tropicalis*	F: GCGGTAGGAGAATTGCGTT R:TCATTATGCCAACATCCTAGGTTTA P: FAM-CGCAGTCCTCAGTCTAGGCTGGCAG-TAMRA	28SrRNAD1/D2 region
Duplex 3		
* Candida krusei*	F:GCT GCG ACT CGC CTG AA R:TTG TCT CGC AAC ACT CGC TCT P:VIC-CTA GTT CGC TCG GCC AGC TTC GCT-TAMRA	ITS2
* Candida parapsilosis*	F:GGG TTT GGT GTT GAG CGA TAC R:GGA GTT TGT ACC AAT GAG TGG AAA P: FAM-CTC CGC CTT TCT TTC AAG CAA ACC CAG-TAMRA	ITS2

^a^*F*: forward, *R*: reverse, *P*: probe

^b^ITS: internal transcribed spacer

### Internal transcribed spacer (ITS) sequencing

ITS sequencing was used as a gold standard to identify *Candida* spp.. Total DNA was isolated from *Candida* isolates and amplified using ITS1/4 primers. Shanghai Biological Engineering Technology Co., Ltd. sequenced the amplicons, edited them with the BioEdit Sequence Alignment program, and compared them to reference ITS regions deposited in the GenBank Database.

### Statistical analysis

Statistical analysis was performed using software GraphPad Prism 7.0. Descriptive statistics, including means and standard deviations, were used to summarize continuous measures. For continuous variables, independent t-tests were applied. Real-time PCR efficiency, statistical significance between slops and intercepts were calculated through linear regression analysis. P < 0.05 was considered significant.

## Results

### Determine the optimal combination use of digestive enzymes

Table 2 shows the lysis efficiency of various digestive enzymes and their combinations (for details, see Additional file 1: Table S1–S6 and Additional file 2: Figure S1–S5 in supplemental materials). The snailase and lyticase could effectively lyse the blastospores of *C. albicans*. The blastospore lysis rates were steadily raised in line with the increasing doses of snailase and lyticase. According to the findings, 1040 μg snailase or 200 U lyticase could disrupt 5 × 10^7^ CFUs of the blastospores of *C. albicans* in 1 mL lysis solution. The enzymes glucanase and zymolyase showed no effect on blastospore lysis. Following that, a combination of snailase and lyticase was used to achieve higher lysis efficient. Dose dependent studies were performed to determine the optimal concentrations for the combination use of lyitcase and snailase. It was confirmed that 650 μg snailase combined with 100 U lyticase could completely disrupt 5 × 10^7^ CFUs of *C. albicans* blastospores in 1 ml lysis solution. Correspondingly, 130 μg snailase combined with 20 U lyticase could totally disrupt 5 × 10^5^ CFUs of *C. albicans* blastospores in 1 h.

**Table 2:** Lysis efficiency of digestive enzymes and their combinations against *Candida albicans* SC5314

Number of blastospores	Combination use	Digestive enzyme	Digestion time (h)	Dosage^a^
5 × 10^7^	No	Lyticase	5	200 U
5 × 10^7^	No	Snailase	5	1 040 μg
5 × 10^7^	No	Zymolyase	5	–
5 × 10^7^	No	Glucanase	5	–
5 × 10^7^	Yes	Lyticase	1	650 μg
	Yes	Snailase	1	100 U
5 × 10^5^	Yes	Lyticase	1	130 μg
	Yes	Snailase	1	20 U

“-”means no obvious effect on SC5314 blastospores

^a^enzyme dosage used in 1 mL system that could completely lyse specified number of blastospores

### PCR inhibitors in the lysis solution

The interference experiment displays the Ct values under different conditions using purified SC5314 DNA as the template. As shown in Table 3, the principal chemicals that interfere with PCR are sorbitol and EDTA. When 12.5 mM sorbitol or 0.625 mM EDTA were added to the PCR reaction, no amplification curve was observed, which was identical to the negative control. While in the presence of 16 µg snailase, 2.5 U lyticase or 0.05 µL β-mercaptoethanol, the Ct values were identical to those obtained from the positive control.

**Table 3:** Impact of the lysis solution components on PCR amplification

Test NO^a^	Lysate components	Amount(/50 µL)^b^	Templates	Ct values^c^
P	–	–	*C. albicans DNA*	17.36
N	–	–	–	–
1	snailase	16 µg	*C. albicans DNA*	17.37
2	lyticase	2.5 U	*C. albicans DNA*	17.36
3	β-mercaptoethanol	0.05 µL	*C. albicans DNA*	17.28
4	sorbitol	12.5 mM	*C. albicans DNA*	–
5	EDTA	0.625 mM	*C. albicans DNA*	–

^a^*P* positive control, *N* negative control

^b^The amounts were calculated according to the optimized lysis solution components

^c^The Ct (crossing threshold) values describe the point where the amplification curve exceeds the noise band

### Optimization of the lysis solution

Removing sorbitol and EDTA from the lysis solution had no effect on lysis ability when co-incubated with SC5314 blastospores. A 100 μL lysis solution containing 130 μg snailase, 20 U lyticase and 1 μL β-mercaptoethanol could totally disrupt 5 × 10^5^ CFUs of SC5314 blastospores under 30 °C for 1 h. Further investigation indicated that among the five *Candida* spp. investigated, the lysis solution could successfully lyse blastospores of *C. albicans*, *C. tropicalis* and *C. parapsilosis*. The blastospores of *C. glabrata* and *C. krusei*, on the other hand, appear to stay mostly intact even when the enzyme concentration was raised significantly (shown in Additional file 2: Figure S7).

### High-temperature metal bath improved the PCR amplification effect

**Fig. 1:**
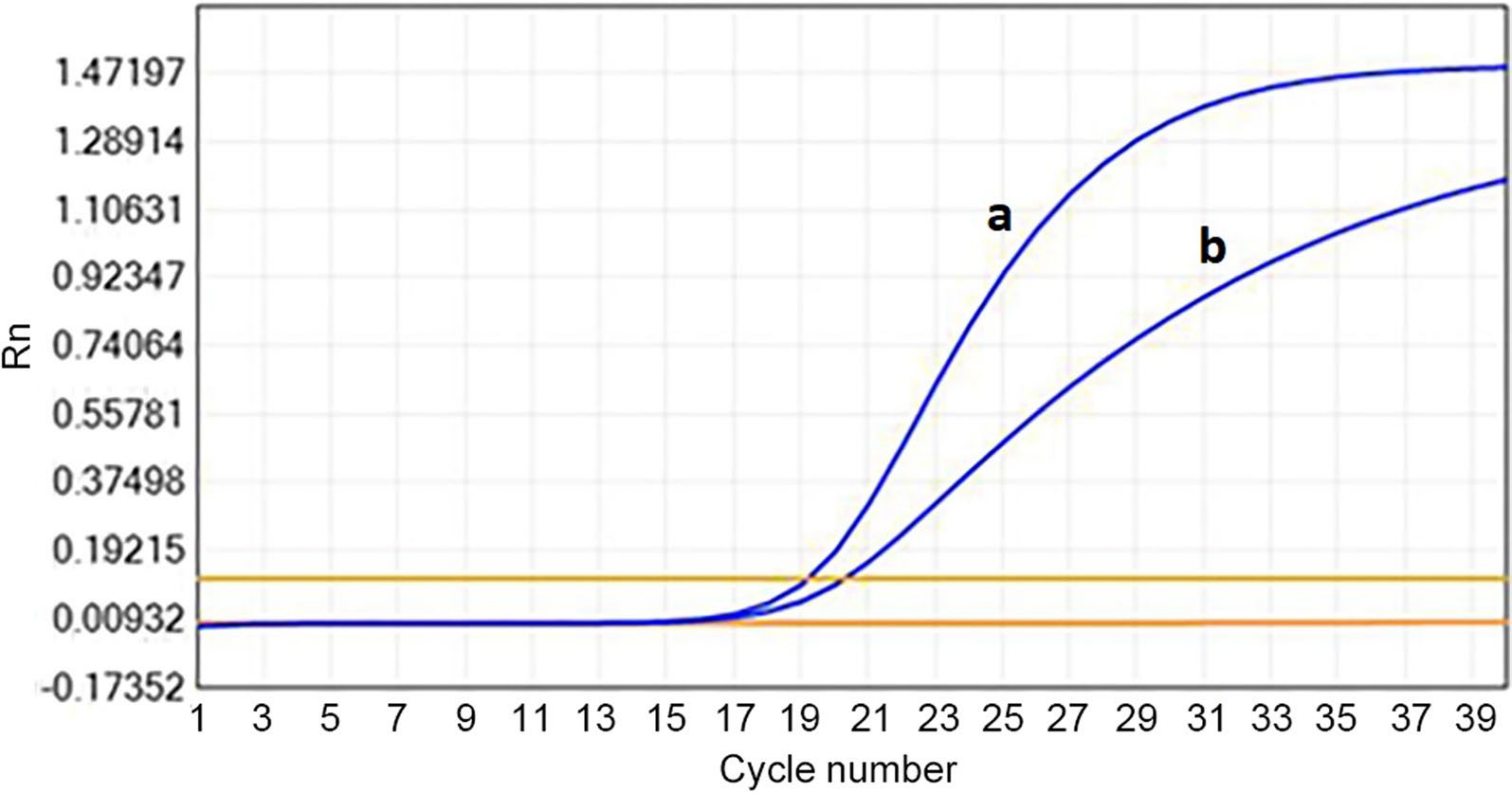
Better PCR amplification effect was obtained after enzyme-lysed *C.albicans* sample treated with high temperature metal bath. The amplification curve produced by samples treated with high temperature metal bath (a), which displays a typical S-shape with significantly higher ΔRn and lower Ct values when comparing with the curve produced by the sample without metal bath treatment (b)

As shown in Fig. 1, PCR on high-temperature metal bath treated *C. albicans* lysates produced a typical S-shaped amplification curve with significantly higher ΔRn (1.35 ± 0.03 VS 1.06 ± 0.02) values and lower Ct values (19.08 ± 0.06 VS 20.26 ± 0.11) when compared to untreated samples.

### Sensitivity and specificity of the duplex PCR

Five *Candida* spp.’s cultured blastospores were collected and adjusted to indicate densities. The samples were treated with a high temperature metal bath after being incubated with the optimized lysis solution. When lysate supernatants were used as templates for PCR amplification, the sets of specific primers and probes were able to effectively amplify the corresponding *Candida* samples, with detection limits as low as 10^0^ CFUs (shown in Additional file 2: Figure S8). The detection limits of *Candida* universal primers (ITS1 and ITS4) were varied when detecting different *Candida* species, which was 10^1^ CFUs for *C. glabrata* and *C. parapsilosis* and 10^0^ CFUs for *C. albicans*, *C. tropicalis*, and *C. krusei* (shown in Additional file 2: Figure S9).

The cross-amplification of primers and probes with non-*Candida* pathogens was also assessed. The non-*Candida* samples were included a variety of clinically prevalent bacterial isolates and sera from various virus-infected individuals. As shown in Table 4 and Additional file 2: Figure S10, the duplex PCR assay correctly identified the corresponding *Candida* spp., with no cross-reaction with DNA prepared from other pathogens.

**Table 4:** Species tested by the duplex PCR assay

Species	Sample	Identification method^a^	Duplex PCR result
*Candida albicans* SC5314 (ATCCMYA-2876)	Standard strain	–	*Candida albicans*
*Candida tropicalis* ATCC1369	Standard strain	–	*Candida tropicalis*
*Candida glabrata* ATCC15126	Standard strain	–	*Candida glabrata*
*Candida parapsilosis* ATCC22019	Standard strain	–	*Candida parapsilosis*
*Candida krusei* ATCC6258	Standard strain	–	*Candida krusei*
*Saccharomyces cerevisiae*ATCC9763	Standard strain	–	Negative
*Escherichia coli*(n = 3)	Clinical isolates	Biochemical	Negative
*Klebsiella pneumoniae*(n = 3)	Clinical isolates	Biochemical	Negative
*Pseudomonas aeruginosa*(n = 3)	Clinical isolates	Biochemical	Negative
*Acinetobacter baumannii*(n = 3)	Clinical isolates	Biochemical	Negative
*Enterococcus faecium*(n = 3)	Clinical isolates	Biochemical	Negative
*Enterococcus faecalis*(n = 3)	Clinical isolates	Biochemical	Negative
*Streptococcus pneumoniae*(n = 3)	Clinical isolates	Biochemical	Negative
*Staphylococcus aureus*(n = 3)	Clinical isolates	Biochemical	Negative
*Staphylococcus epidermis*(n = 3)	Clinical isolates	Biochemical	Negative
Group B *streptococci*(n = 3)	Clinical isolates	Biochemical	Negative
*Hepatitis* B virus(n = 3)	Positive serum	PCR	Negative
*Hepatitis* C virus(n = 3)	Positive serum	PCR	Negative
*Epstein-Barr* virus(n = 3)	Positive plasma	PCR	Negative

^a^Biochemical identification was completed with a MA120 automated ID &Ast system (Meihua Med Tech, Zhuhai, China) in an ISO 9000 accredited laboratory

### Comparing the rapid DNA preparation method with conventional method

We evaluate the PCR amplification impact of our DNA preparation method with another method that used lyticase and a DNA purification kit (Ma 2009). There were clear differences between the PCR Ct values of the five different *Candida* spp. DNA templates obtained by the two methods at a gradient blastospore load ranging from 10^0^ to 10^5^ CFUs. The DNA template obtained from our method yielded lower Ct values in the five *Candida* species investigated (for details, see Additional file 1: Table S7–S11). Statistical analysis revealed that the y-intercept of linear regression for PCR Ct values obtained by different DNA preparation methods differed significantly. The rapid DNA preparation method provided a lower intercept of linear regression than the conventional method (Table 5).

**Table 5:** Comparison of linear regression parameters for PCR Ct values obtained from different DNA preparation methods

Species	Slopes (95% CI)^a^		Significance^b^ (P value)	y-Intercepts (95% CI)^a^		Significance^b^ (P value)
	Conventional	Rapid		Conventional	Rapid	
*Candida albicans*	− 3.125 (− 3.601 to − 2.648)	− 3.287 (− 3.414 to -3.16)	0.3875	37.59 (36.15 to 39.04)	32.65 (32.26 to 33.03)	< 0.0001^*^
*Candida tropicalis*	− 2.604 (− 3.076 to − 2.132)	− 2.806 (− 3.362 to − 2.25)	0.4641	36.01 (34.58 to 37.44)	30.43 (28.75 to 32.11)	< 0.0001^*^
*Candida glabrata*	− 2.860 (− 3.519 to − 2.202)	− 3.119 (− 3.38 to − 2.859)	0.3391	37.05 (35.06 to 39.04)	34.35 (33.56 to 35.14)	< 0.0001^*^
*Candida parapsilosis*	− 2.968 (− 3.44 to − 2.497)	− 2.973 (− 3.268 to − 2.677)	0.9835	37.89 (36.46 to 39.32)	32.37 (31.48 to 33.27)	< 0.0001^*^
*Candida krusei*	− 3.643 (− 4.033 to − 3.252)	− 3.355 (− 3.518 to − 3.192)	0.0961	38.35 (37.17 to 39.53)	32.39 (31.9 to 32.88)	< 0.0001^*^

^a^The comparison was performed between two different DNA preparation methods, “conventional” here represents the conventional DNA preparation method, and “rapid” represents the rapid DNA preparation method we established

^b^P values were calculated by default statistical method of the software GraphPad Prism 7.00

^*^Statistically significant (p < 0.05)

### Comparing PCR with microbial culture for clinical isolate identification

A total of 30 biochemically identified *Candida* clinical isolates were collected and subjected to duplex PCR and ITS sequencing in this study (He et al. 2021). Table 6 and Additional file 1: Table S12 illustrate the results. Except for one *C. parapsilosis* isolate that was misidentified as *C. Krusei* by duplex PCR and ITS sequencing, the results of microbial culture, duplex PCR and ITS sequencing were completely concordant for all of the remaining *Candida* isolates included. The overall consistency for the three identification methods was 96.67%, with duplex PCR and ITS sequencing being 100% consistent.

**Table 6:** The identification results of PCR, microbial culture and ITS sequencing for the 30 *Candida* clinical isolates

Microbial culture identified isolates (n)	ITS sequencing			PCR	
	Yes	No		Yes	No
*C. albicans*(5)	5	0		5	0
*C. tropicalis*(6)	6	0		6	0
*C. glabrata*(6)	6	0		6	0
*C. parapsilosis*(7)	6	1^a^		6	1^a^
*C. krusei*(6)	6	0		6	0

^a^One isolate identified as *C. parapsilosis* by microbial culture was identified as *C. krusei* both by ITS sequencing and PCR

## Discussion

Rapid species identification methods including PCR are becoming increasingly important for clinical control of *Candida* infection due to the fact that the incidence rate of IC is sustained high in recent years. Since the time-consuming steps of DNA extraction and purification were necessary, conventional PCR methods for *Candida* spp. detecting usually take 6 h or more (Zhang et al. 2016). Furthermore, the likehood of DNA loss and tedious operation are notable limitations for typical DNA extraction methods when used in clinical labs with high sample flow (Dalla-Costa et al. 2017). In this study, we provide a rapid and bio-safe *Candida* DNA preparation method for PCR detection that takes only about 3 h from sample collection to obtain the results.

DNA sample preparation method is one of the key factors that affecting PCR identification for *Candida* species (Dalla-Costa et al. 2017). As previously described (Kim et al. 2016; Mazoteras et al. 2015; Rickerts et al. 2012), enzyme digestion and high temperatures are efficient ways for releasing *Candida* DNA. To achieve higher lysis efficiency, we combined two enzymes. Contrary to our expectation, the combined enzyme solution was able to lyse *C. albicans*, *C. tropicalis* and *C. parapsilosis*, although it had no impact in *C. glabrata* and *C. krusei*. However, the PCR results showed that the incomplete lysis had no effect on the sensitivity of the following amplification after the high temperature metal bath. We hypothesize that the high temperature metal bath that impacted on the rather fragile cell wall that had been treated by digestive enzymes completed the DNA release. Previously, we demonstrated that the sensitivity of PCR could be increased to 10^1^ CFUs utilizing a nucleic acid sample generated solely by the "heat-shock" approach (He et al. 2020). High temperature treatment also inactivates various bioactive components of the fungal lysate, which aids in reducing the influence of crude DNA template on the PCR process (Chen et al. 2019).

The lysed *Candida* supernatant was directly used as a PCR template to reduce the difficulty and labor of DNA sample preparation. To this end, we designed interference experiments to identify and remove the inhibitors, as well as a metal bath to inactivate the protein PCR inhibitors in the samples. The PCR amplification curve of a template prepared by enzyme lysis coupled with a metal bath was of the standard "S" type, which was superior to samples not treated with high temperature. Another key issue with sample processing in clinical labs is bio-safety. The *Candida* DNA preparation method presented in this paper could be completed in a closed test tube and then finally treated in a high-temperature metal bath, considerably reducing the biohazard risk.

**Fig. 2:**
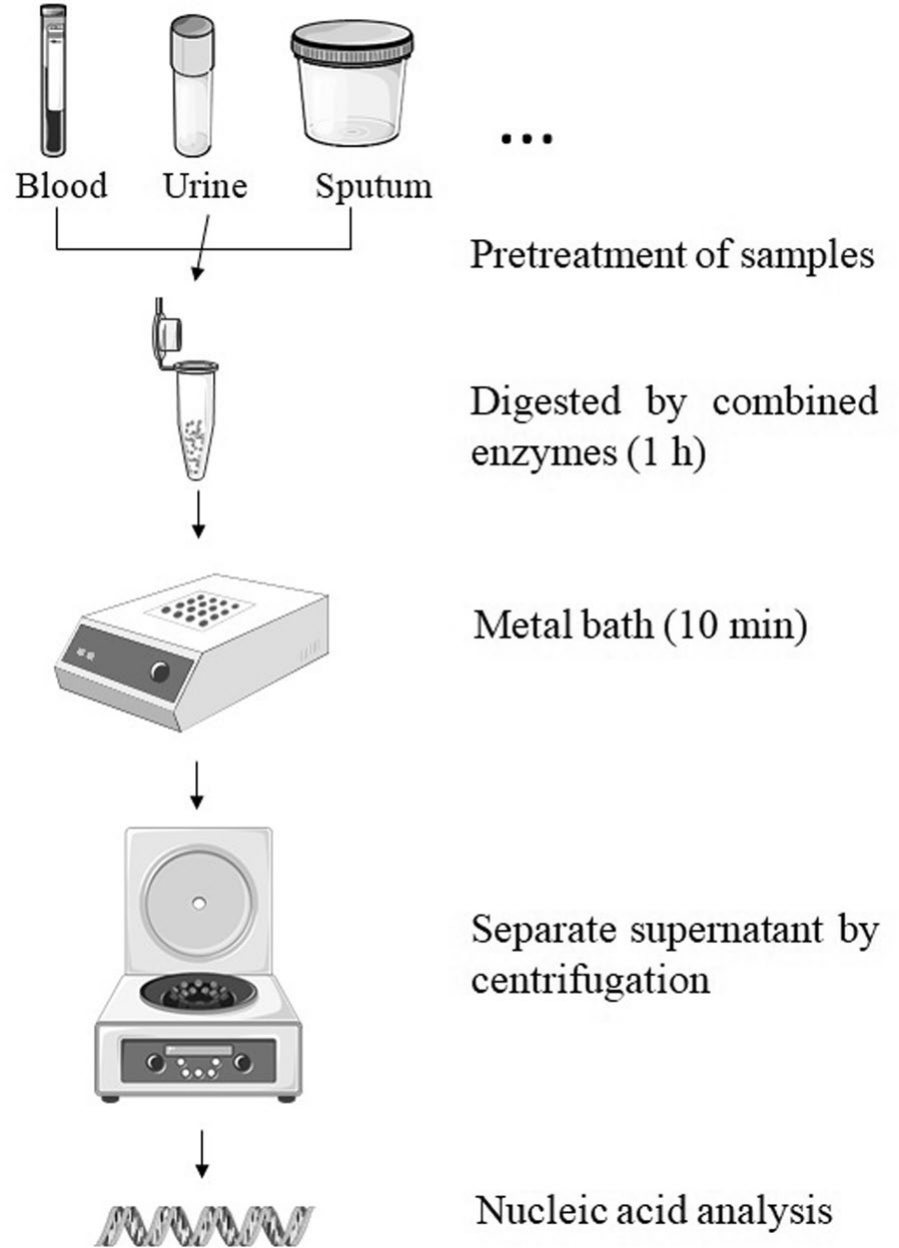
Schematic of the rapid *Candida* DNA preparation and detection workflow. *Candida* blastospores were applied to enzyme digestion and high temperature metal bath after separated from pretreated clinical samples. After centrifugation, the supernatants were directly used as templates for nucleic acid analysis. The metal bath instrument was drawn by JP, and the other figure elements were taken from the free medical image site at https://smart.servier.com/

Our study so far suggests a general experimental workflow for preparing *Candida* nucleic acid samples as indicated by Fig. 2. After simple pretreatment for different types of clinical samples (eg, sputum, urine or whole blood), the precipitated *Candida* cells were digested by the lysis solution for 1 h and treated with a high temperature metal bath for 10 min to fully release the *Candida* DNA. After centrifugation, the supernatant can be directly used as a template for further nucleic acid analysis.

Five sets of specific and one set of universal primers/probes were used to identify common clinical *Candida* spp.. The PCR sensitivity of the specific primers/probes to their corresponding *Candida* species was 10^0^ CFUs, which was higher than that assessed by Foongladda et al. (Foongladda et al. 2014). The sensitivity of the universal primers/probes ranged from 10^0^ to 10^1^ CFUs, yielding superior results than previous studies using the identical primers/probes (Guo et al. 2016). Although the improved sensitivity may be due to various PCR reagents and the settings of the amplification, the higher DNA yield of the preparation method described in this work may be a more relevant reason.

We selected 30 *Candida* clinical isolates to evaluate the diagnosis accuracy of PCR using ITS sequencing as the gold standard. The PCR and ITS sequencing were completely consistent. It is worth noting that a strain of *C. krusei* validated by ITS sequencing was misidentified as *C. parapsilosis* by microbial culture. Among the possible causes are: (i) the incidence rate of *C. albicans* and *C. tropicalis* infection is relatively high, and the detection system for them is mature (Xiao et al. 2018); (ii) the CHROMagar coloration and biochemical reaction characteristics are clear for *C. albicans* and *C. tropicalis*, but not for some *C. parapsilosis*, *C. glabrata* and *C. krusei* isolates (Jafari et al. 2017).

It should be mentioned that, in a variety of nucleic acid detection methods, this study only employed PCR as a representative to validate the rapid *Candida* DNA preparation method. Other detection methods, such as the LAMP technique, are capable of detecting the *Candida* species with high sensitivity and specificity, without the need for expensive instruments (Fallahi et al. 2020). Kasahara et al. devised a multiplex LAMP test with a detection limit of 10^0^ CFU/mL for medically important yeasts (Kasahara et al. 2014). Hence, combining the rapid DNA preparation method and other nucleic acid detection methods might provide a feasible and easy protocol for *Candida* detection in the future.

Here we present a novel method of rapid *Candida* DNA preparation that has the benefits of high DNA yield, simple operation, easy standardization, and bio-safety. When combined with the duplex PCR test, the rapid preparation method might have practical utility in accurately diagnosing *Candida* infection in the clinical laboratories. Meanwhile, the practical application of the method for clinical samples needs to be proven in future investigations.

## Supplementary Information

**Additional file 1: Table S1.** Digestive effect of snailase on Candida albicans blastospores. **Table S2.** digestive effect of lyticase on Candida albicans blastospores. **Table S3.** Digestive effect of glucanase on Candida albicans blastospores. **Table S4.** Digestive effect of zymolyase on Candida albicans blastospores. **Table S5.** combination use of snailase & lyticase on Candida albicans blastospores. **Table S6.** combination use of snailase & lyticase on Candida albicans blastospores. **Table S7.** Candida albicans. Table S8. Candida tropicalis. **Table S9.** Candida glabrata. **Table S10.** Candida parapsilosis. Table S11. Candida krusei. **Table S12.** The identification results of PCR, microbial culture and ITS sequencing for the 30 Candida clinical isolates.

**Additional file 2:  Figure S1.** Digestive effect of lyticase on *Candida albicans *(400×). (A)negative control, (B)130µg, (C)650µg, (D)1040µg, (E)2080µg. **Figure S2.** Digestive effect of snailase on *Candida albicans *(400×). (A)negative control, (B)100U, (C)200U, (D)500U, (E)800U. **Figure S3.** Digestive effect of zymolyase on *Candida albicans *(400×). (A)negative control, (B) 5U, (C) 20U, (D) 50U, (E) 100U. **Figure S4.** Digestive effect of glucanase on *Candida albicans *(400×). (A)negative control, (B) 40U, (C) 80U, (D) 160U, (E) 320U. **Figure S5.** Combination use of snailase & lyticase (400×). (A)negative control, (B) 13µg snailase+2U Lyticase, (C) 65µg snailase+10U Lyticase, (D) 130µg snailase+20U Lyticase, (E) 650µg snailase+100U Lyticase, (F) 1040µg snailase+200U Lyticase. **Figure S6.** PCR interference experiment that adding different lysis solution components into the 50 µl PCR reaction system. (1) positive control, (2) 16 µg snailase, 2.5U Lyticase, (3) 0.05 µl β-mercaptoethanol, (4) 12.5mM sorbitol, (5) 0.625mM EDTA, (6) negative control. **Figure S7.** Optimized lysis solution composed of snailase, lyticase and β-mercaptoethanol could totally disrupt of blastospores C. albicans (A), C. tropicalis (B) and C. parapsilosis (C). However, has no effect on blastospores of C. glabrata (D) and C. krusei (E). **Figure S8**. Sensitivity of multiple PCR (specific primers and probes). (A) Candida albicans, (B) Candida tropicalis, (C) Candida parapsilosis, (D) Candida glabrata, and (E) Candida krusei. The numbers of 0-5 represent Candida density of 100-105CFU/ml, and NC represents a negative control. **Figure S9.** Sensitivity of multiple PCR (universal primers and probes). (A) *Candida albicans*, (B) *Candida tropicalis*, (C) *Candida parapsilosis*, (D) *Candida *glabrata, and (E) *Candida krusei*. The numbers of 0-5 represent *Candida* density of 10^0^-10^5^CFU/ml, and NC represents a negative control. **Figure S10.** Specificity of duplex PCR. The amplification curve of A-E were: (A) *Candida. albicans*, (B) *Candida. tropicalis*, (C) *Candida. parapsilosis*, (D) *Candida. glabrata*, (E) *Candida. krusei*. (F), The curves by *Candida* universal primer to amplify five common *Candida* species: (1) *Candida. tropicalis*, (2) *Candida. parapsilosis*, (3*) Candida. krusei*, (4) *Candida. albicans*, (5) *Candida. glabrata*.

## Data Availability

All data generated or analyzed during this study are included in this published article and its supplementary information files.

## Abbreviations

DNA: Deoxyribonucleic acid
PCR: Polymerase chain reaction
IC: Invasive candidiasis
qRT-PCR: Quantitative real-time polymerase chain reaction
LAMP: Loop-mediated isothermal amplification
ATCC: American type culture collection
YDP: Yeast extract peptone dextrose
EDTA: Ethylene diamine tetraacetic acid
ITS: Internal transcribed spacer
CFU: Colony forming unit
Ct: Cycle threshold
PBS: Phosphate buffered saline
