# Supplementary material for: Rapid preparation of Candida genomic DNA: combined use of enzymatic digestion and thermal disruption
Source: AMB Express. 2023 Jan 2;13:1. doi: 10.1186/s13568-022-01500-z (PMC9807692; doi:10.1186/s13568-022-01500-z)
Supplement: Supplementary file 2 — Additional file 2: Figure S1. Digestive effect of lyticase on Candida albicans (400×). (A)negative control, (B)130µg, (C)650µg, (D)1040µg, (E)2080µg. Figure S2. Digestive effect of snailase on Candida albicans (400×). (A)negative control, (B)100U, (C)200U, (D)500U, (E)800U. Figure S3. Digestive effect of zymolyase on Candida albicans (400×). (A)negative control, (B) 5U, (C) 20U, (D) 50U, (E) 100U. Figure S4. Digestive effect of glucanase on Candida albicans (400×). (A)negative control, (B) 40U, (C) 80U, (D) 160U, (E) 320U. Figure S5. Combination use of snailase & lyticase (400×). (A)negative control, (B) 13µg snailase+2U Lyticase, (C) 65µg snailase+10U Lyticase, (D) 130µg snailase+20U Lyticase, (E) 650µg snailase+100U Lyticase, (F) 1040µg snailase+200U Lyticase. Figure S6. PCR interference experiment that adding different lysis solution components into the 50 µl PCR reaction system. (1) positive control, (2) 16 µg snailase, 2.5U Lyticase, (3) 0.05 µl β-mercaptoethanol, (4) 12.5mM sorbitol, (5) 0.625mM EDTA, (6) negative control. Figure S7. Optimized lysis solution composed of snailase, lyticase and β-mercaptoethanol could totally disrupt of blastospores C. albicans (A), C. tropicalis (B) and C. parapsilosis (C). However, has no effect on blastospores of C. glabrata (D) and C. krusei (E). Figure S8. Sensitivity of multiple PCR (specific primers and probes). (A) Candida albicans, (B) Candida tropicalis, (C) Candida parapsilosis, (D) Candida glabrata, and (E) Candida krusei. The numbers of 0-5 represent Candida density of 100-105CFU/ml, and NC represents a negative control. Figure S9. Sensitivity of multiple PCR (universal primers and probes). (A) Candida albicans, (B) Candida tropicalis, (C) Candida parapsilosis, (D) Candida glabrata, and (E) Candida krusei. The numbers of 0-5 represent Candida density of 100-105CFU/ml, and NC represents a negative control. Figure S10. Specificity of duplex PCR. The amplification curve of A-E were: (A) Candida. albicans, (B) Candida. [file 13568_2022_1500_MOESM2_ESM.pptx]

## Slide 1
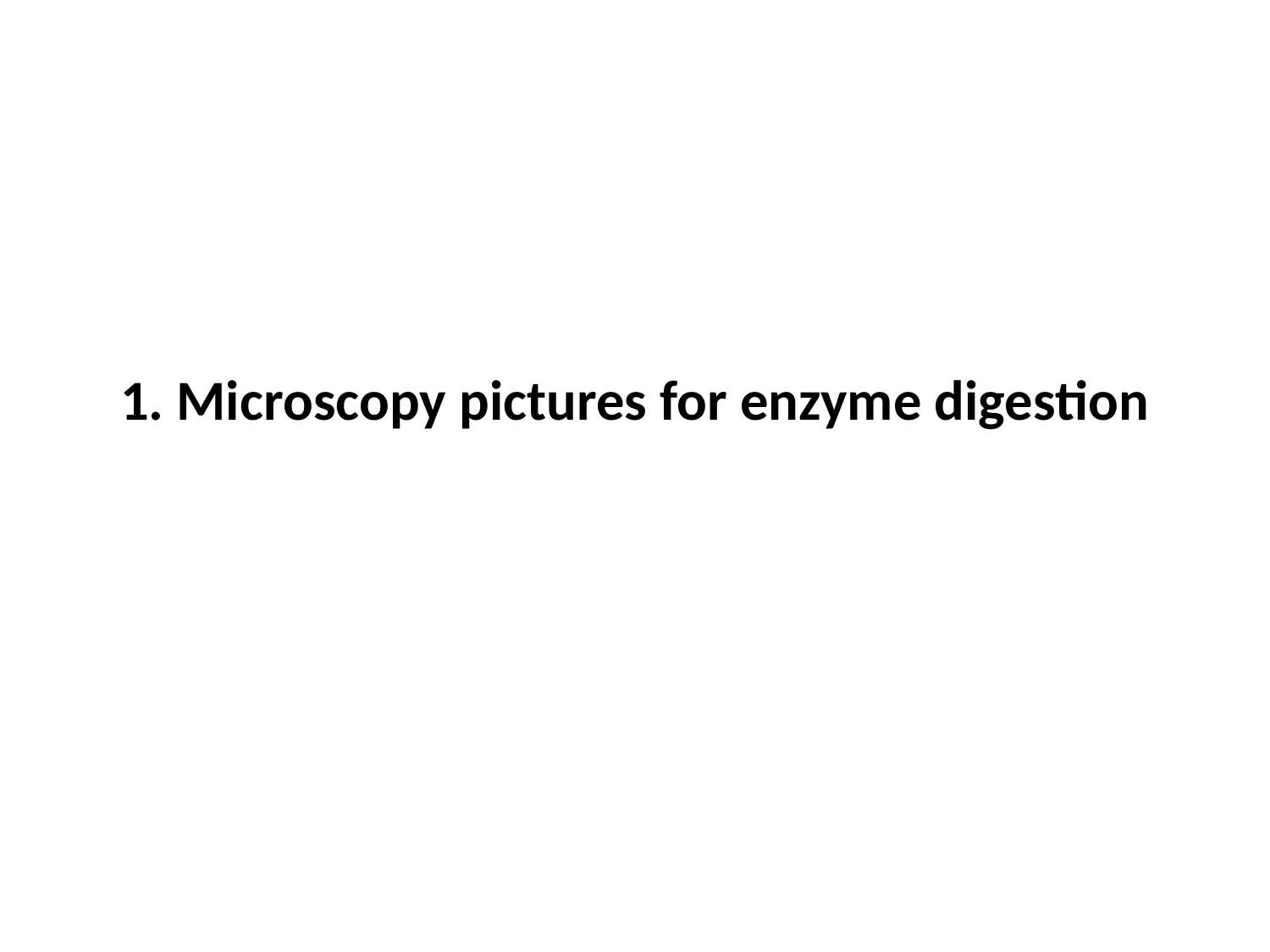

# 1. Microscopy pictures for enzyme digestion

## Slide 2
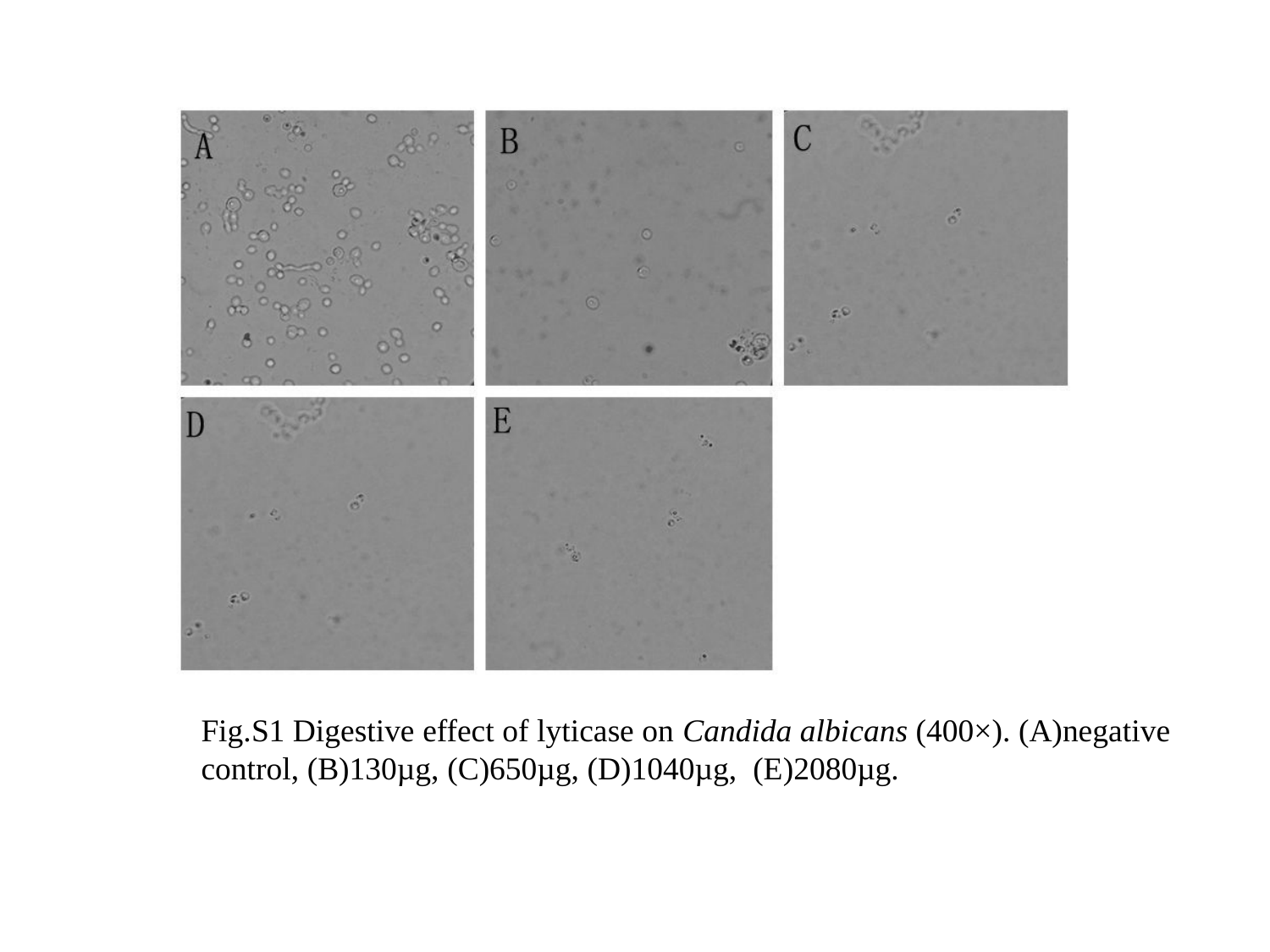

Fig.S1 Digestive effect of lyticase on Candida albicans (400×). (A)negative control, (B)130µg, (C)650µg, (D)1040µg, (E)2080µg.

## Slide 3
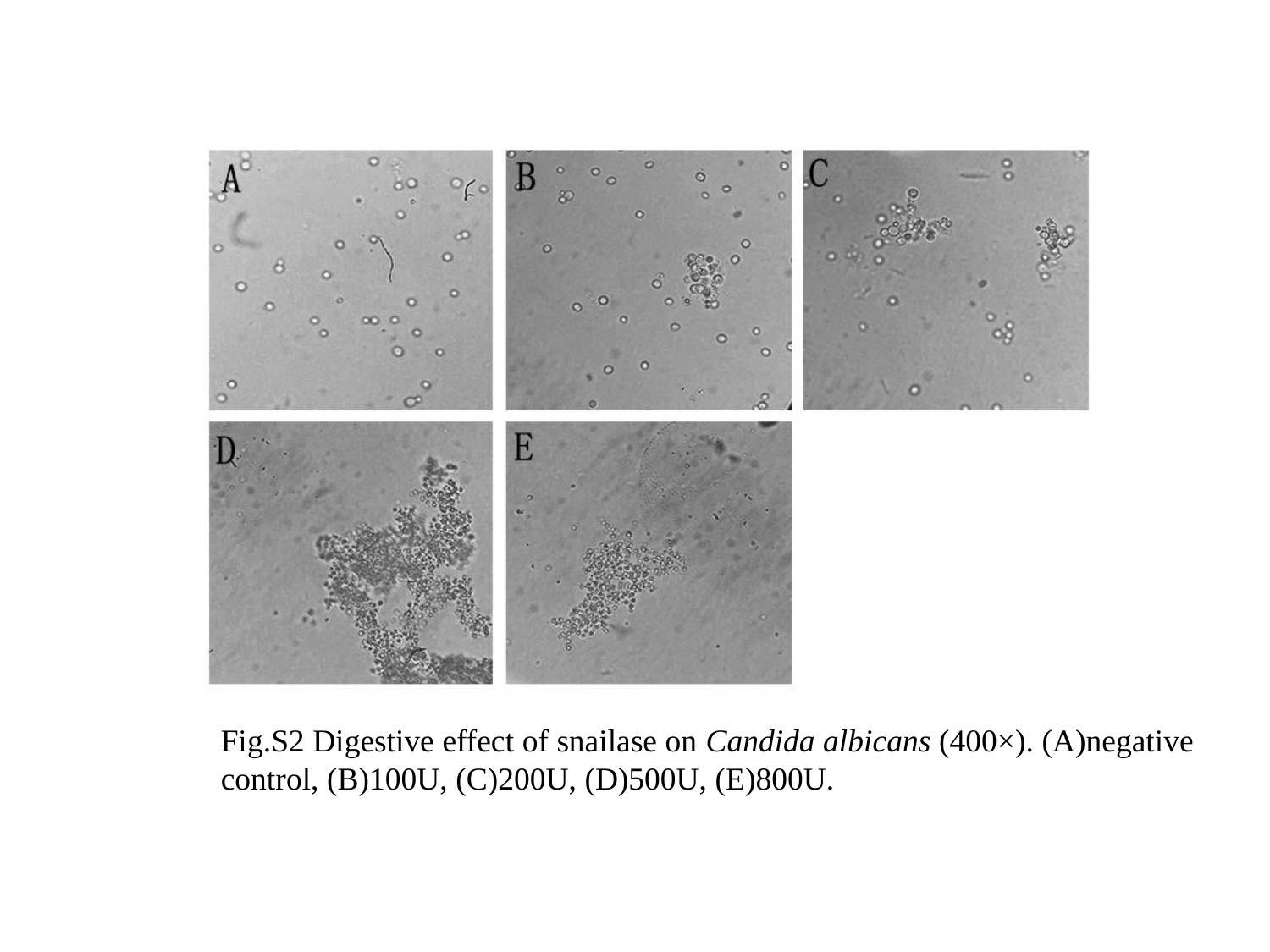

Fig.S2 Digestive effect of snailase on Candida albicans (400×). (A)negative control, (B)100U, (C)200U, (D)500U, (E)800U.

## Slide 4
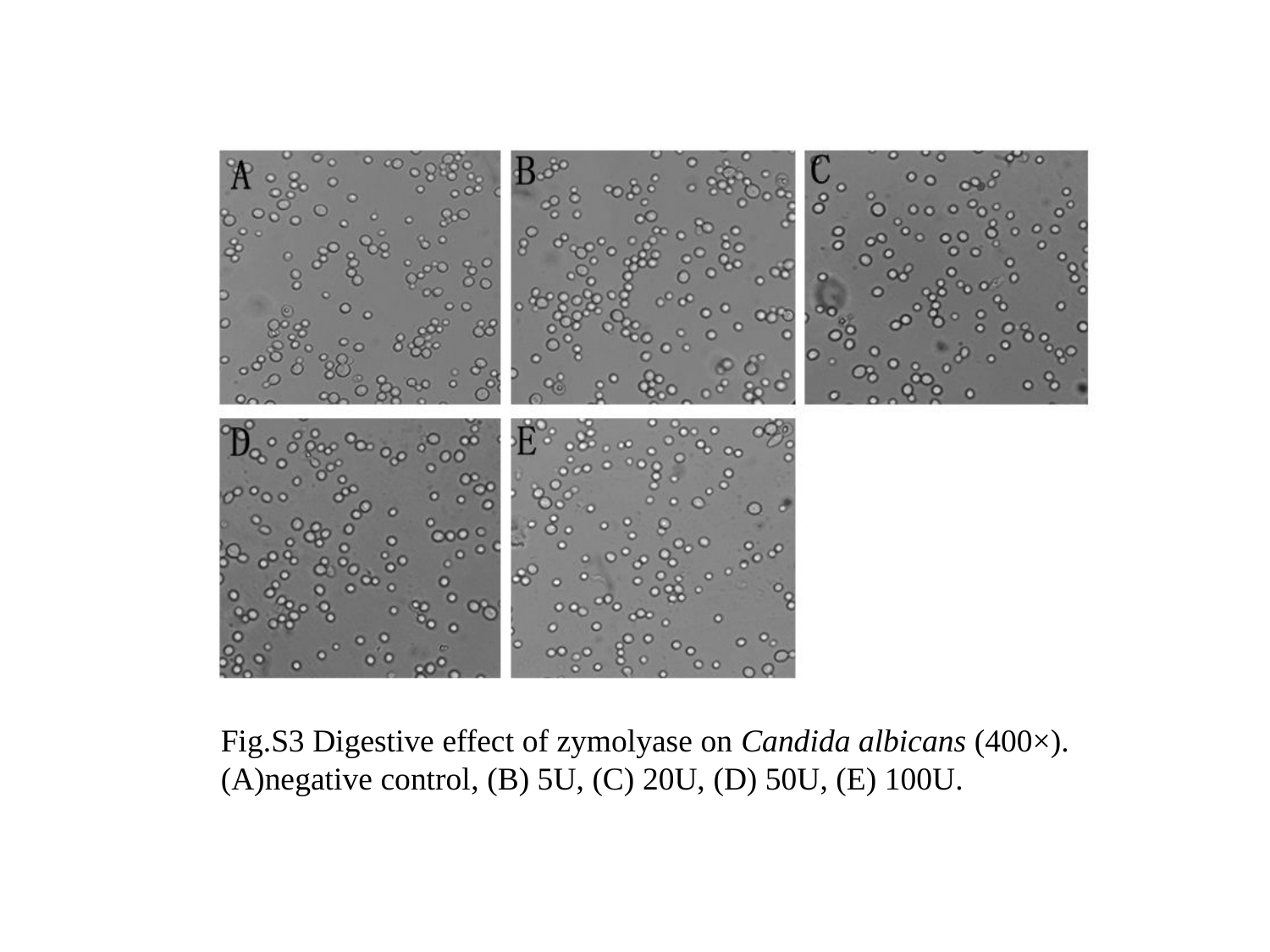

Fig.S3 Digestive effect of zymolyase on Candida albicans (400×). (A)negative control, (B) 5U, (C) 20U, (D) 50U, (E) 100U.

## Slide 5
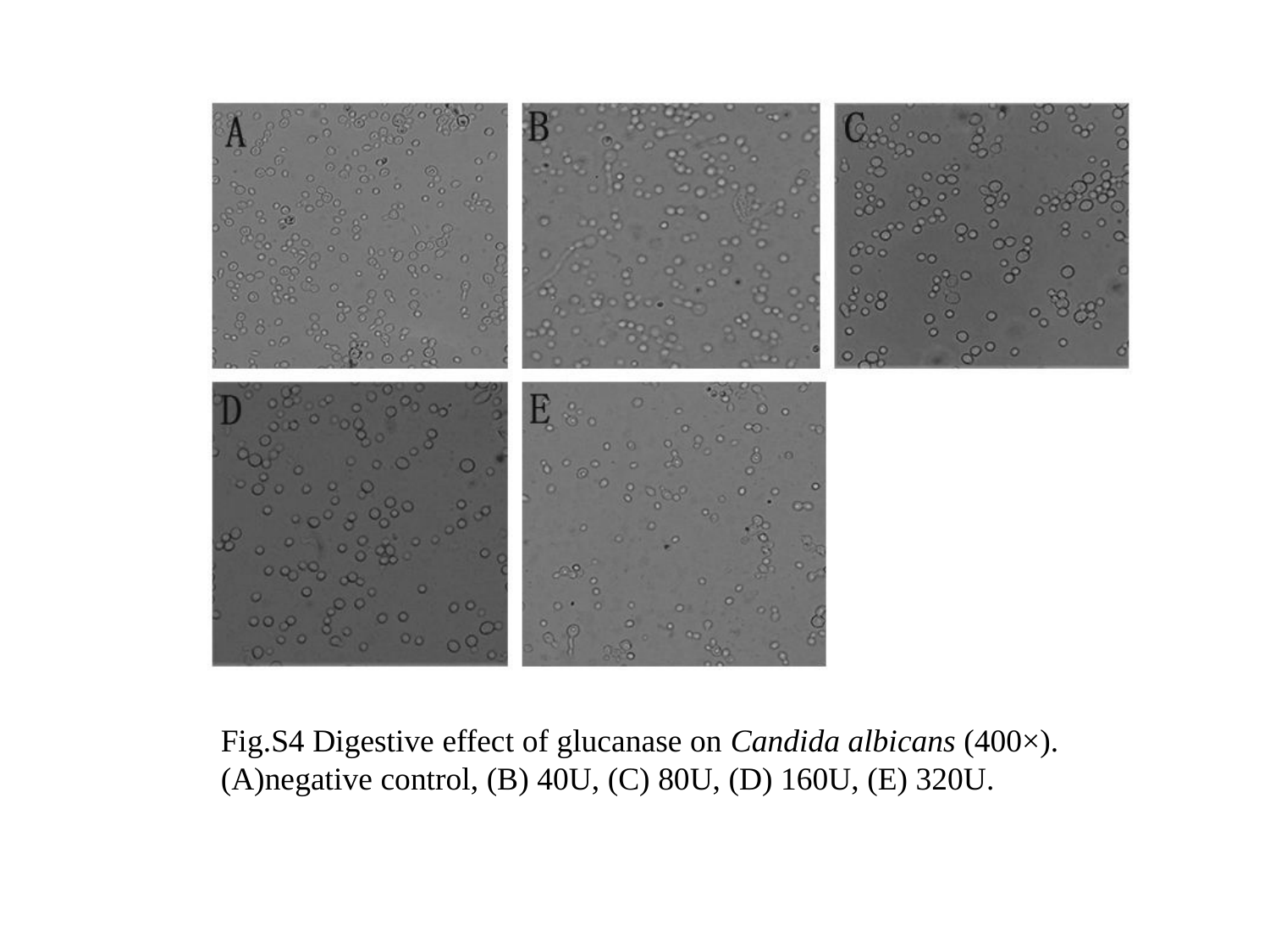

Fig.S4 Digestive effect of glucanase on Candida albicans (400×). (A)negative control, (B) 40U, (C) 80U, (D) 160U, (E) 320U.

## Slide 6
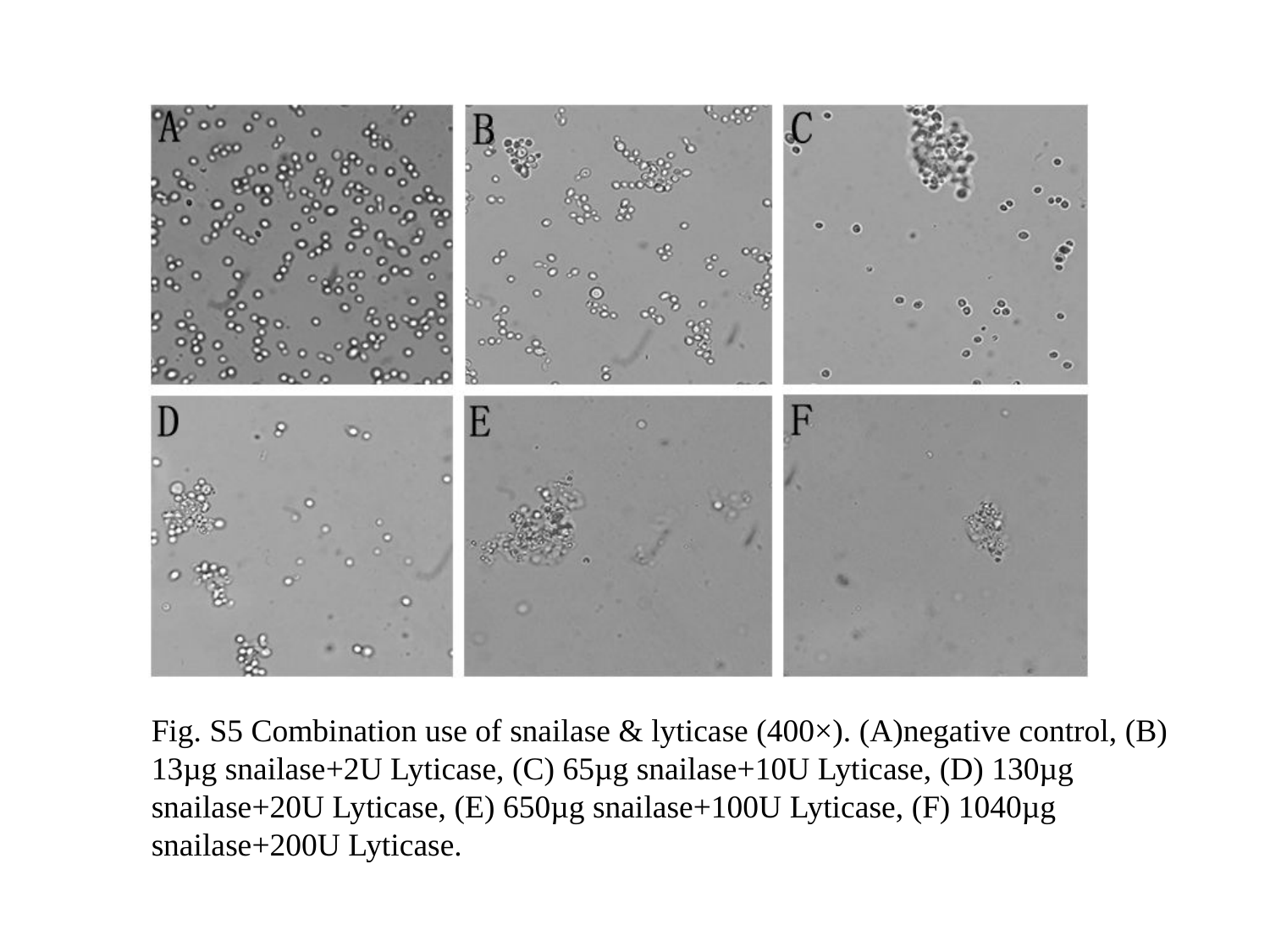

Fig. S5 Combination use of snailase & lyticase (400×). (A)negative control, (B) 13µg snailase+2U Lyticase, (C) 65µg snailase+10U Lyticase, (D) 130µg snailase+20U Lyticase, (E) 650µg snailase+100U Lyticase, (F) 1040µg snailase+200U Lyticase.

## Slide 7
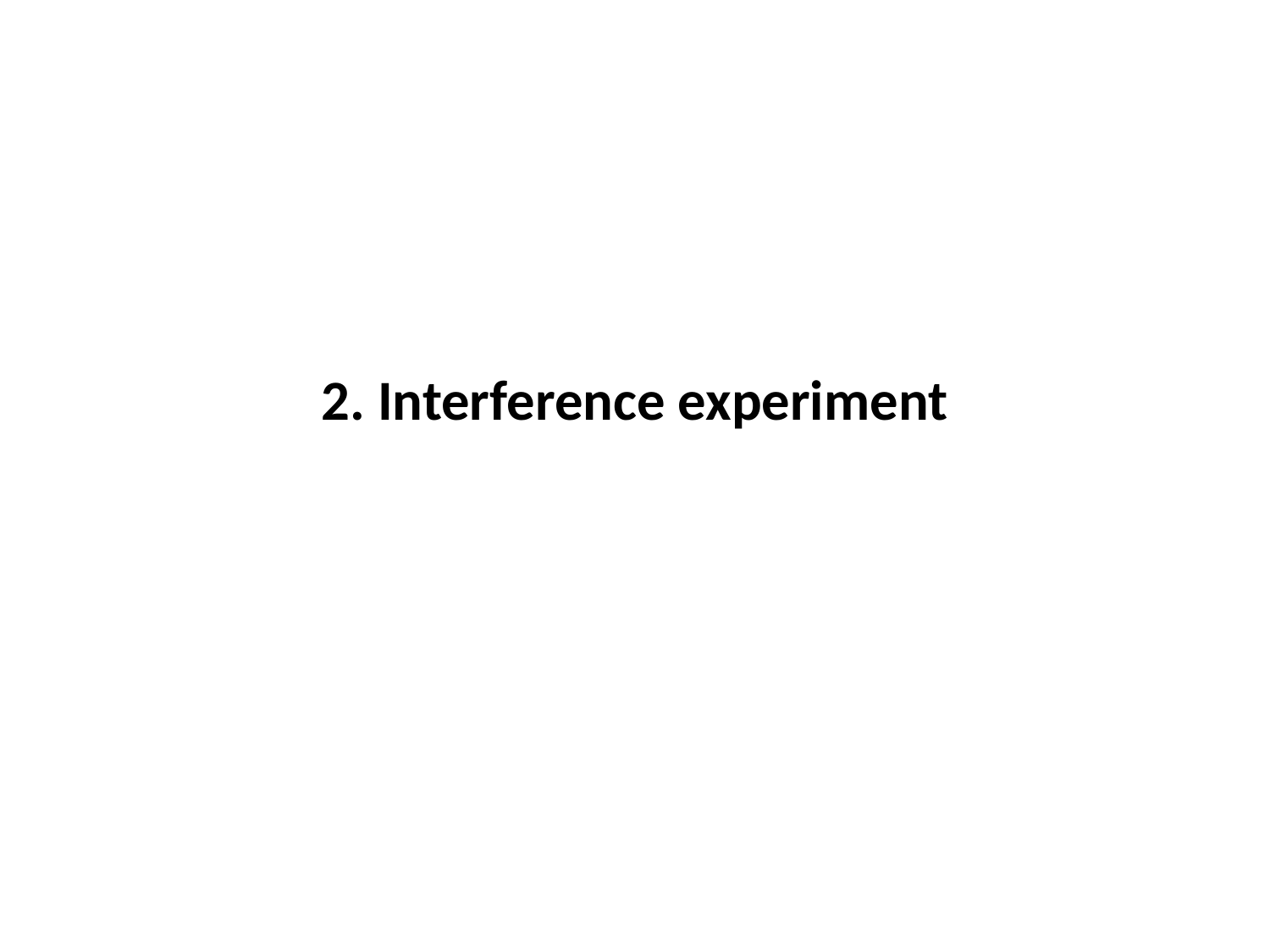

2. Interference experiment

## Slide 8
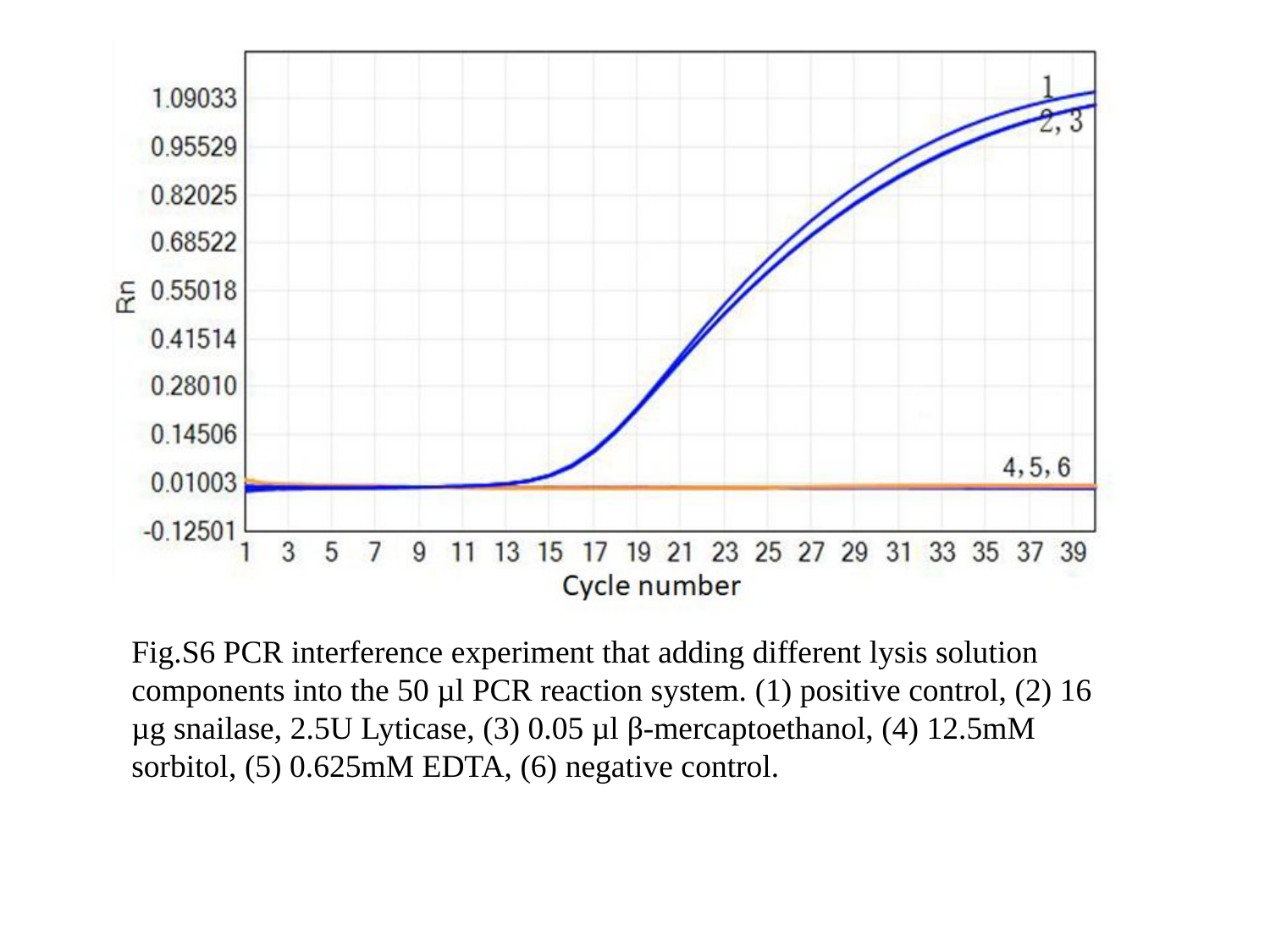

Fig.S6 PCR interference experiment that adding different lysis solution components into the 50 µl PCR reaction system. (1) positive control, (2) 16 µg snailase, 2.5U Lyticase, (3) 0.05 µl β-mercaptoethanol, (4) 12.5mM sorbitol, (5) 0.625mM EDTA, (6) negative control.

## Slide 9
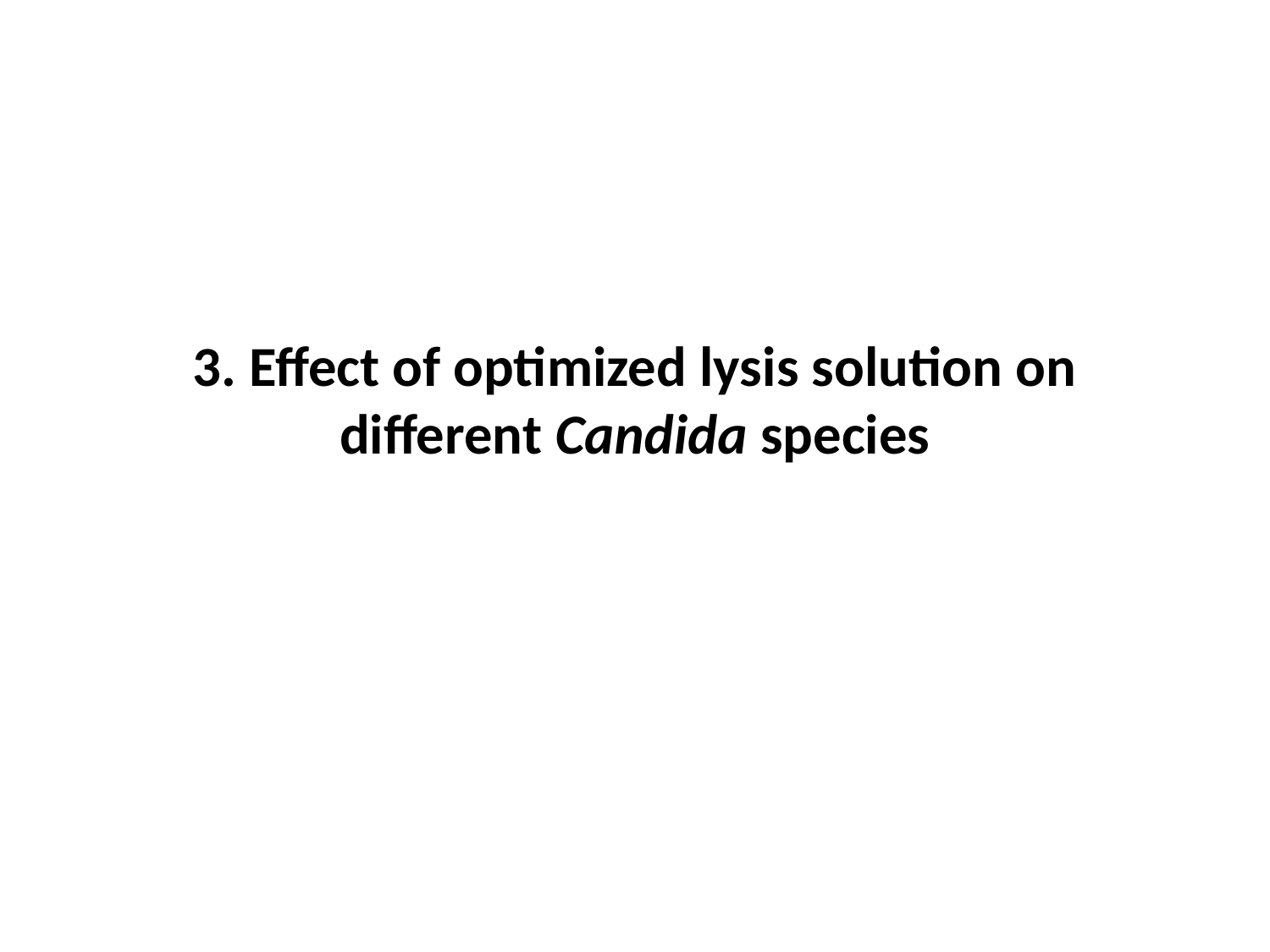

3. Effect of optimized lysis solution on different Candida species

## Slide 10
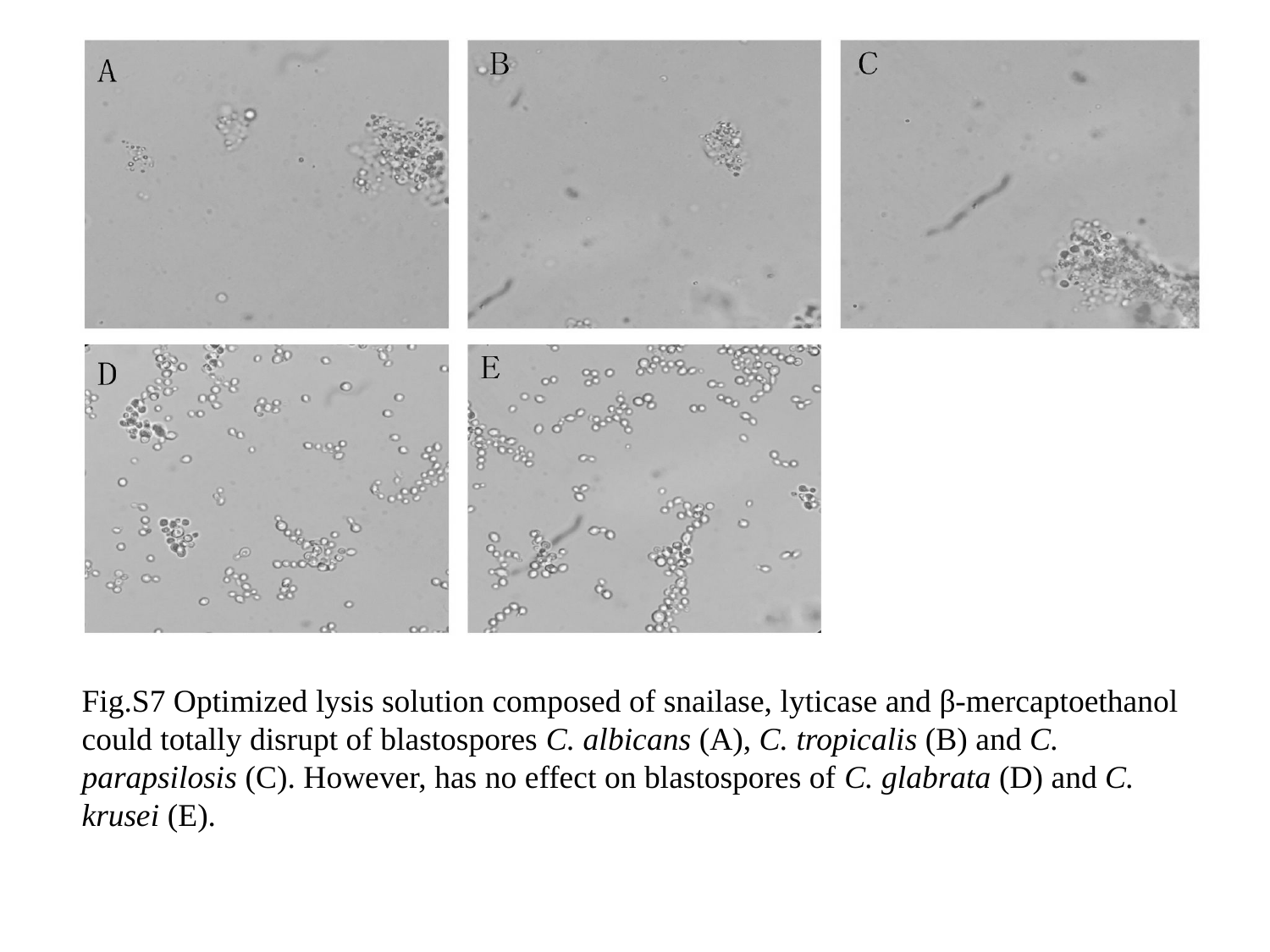

Fig.S7 Optimized lysis solution composed of snailase, lyticase and β-mercaptoethanol could totally disrupt of blastospores C. albicans (A), C. tropicalis (B) and C. parapsilosis (C). However, has no effect on blastospores of C. glabrata (D) and C. krusei (E).

## Slide 11
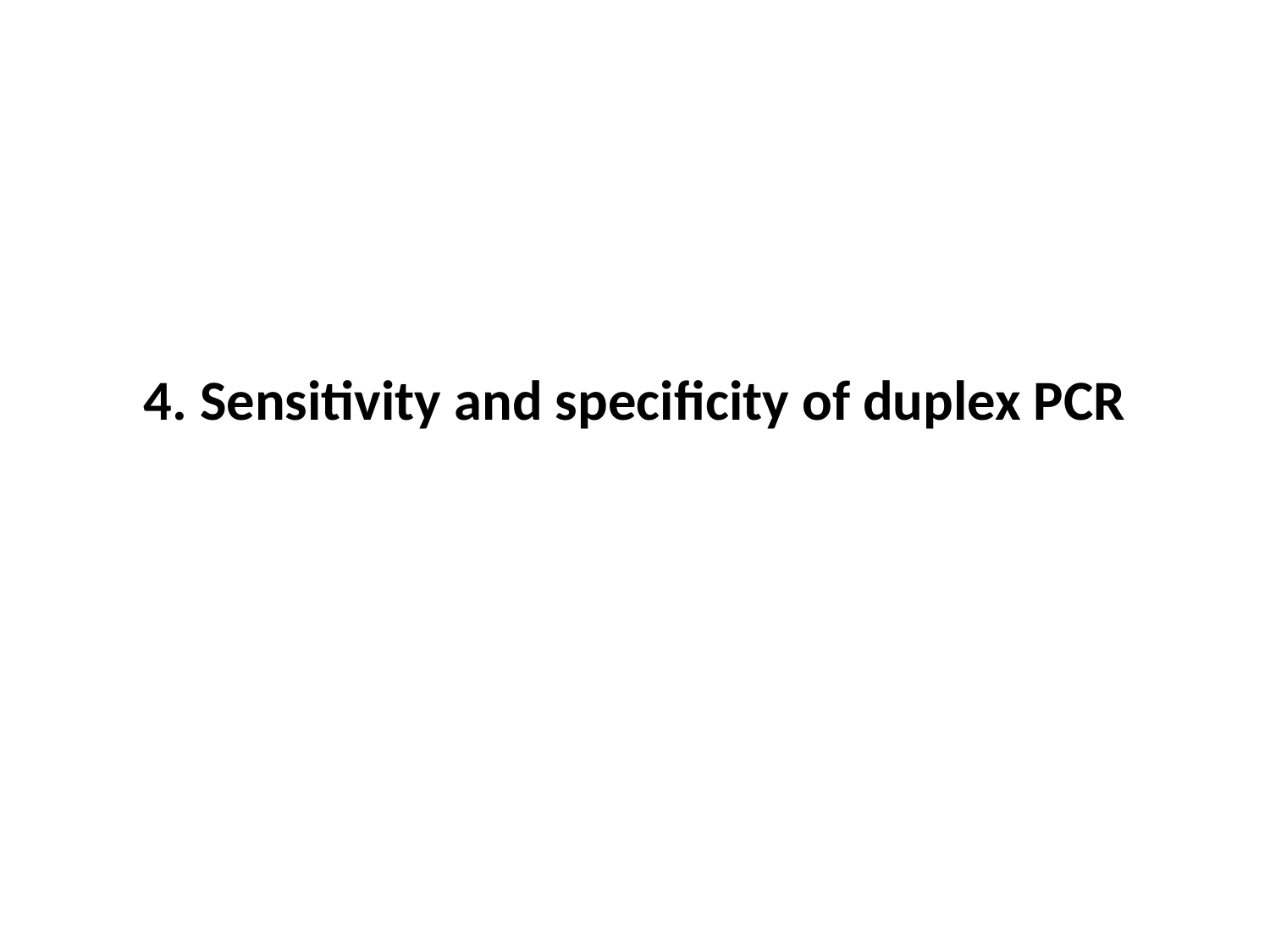

4. Sensitivity and specificity of duplex PCR

## Slide 12
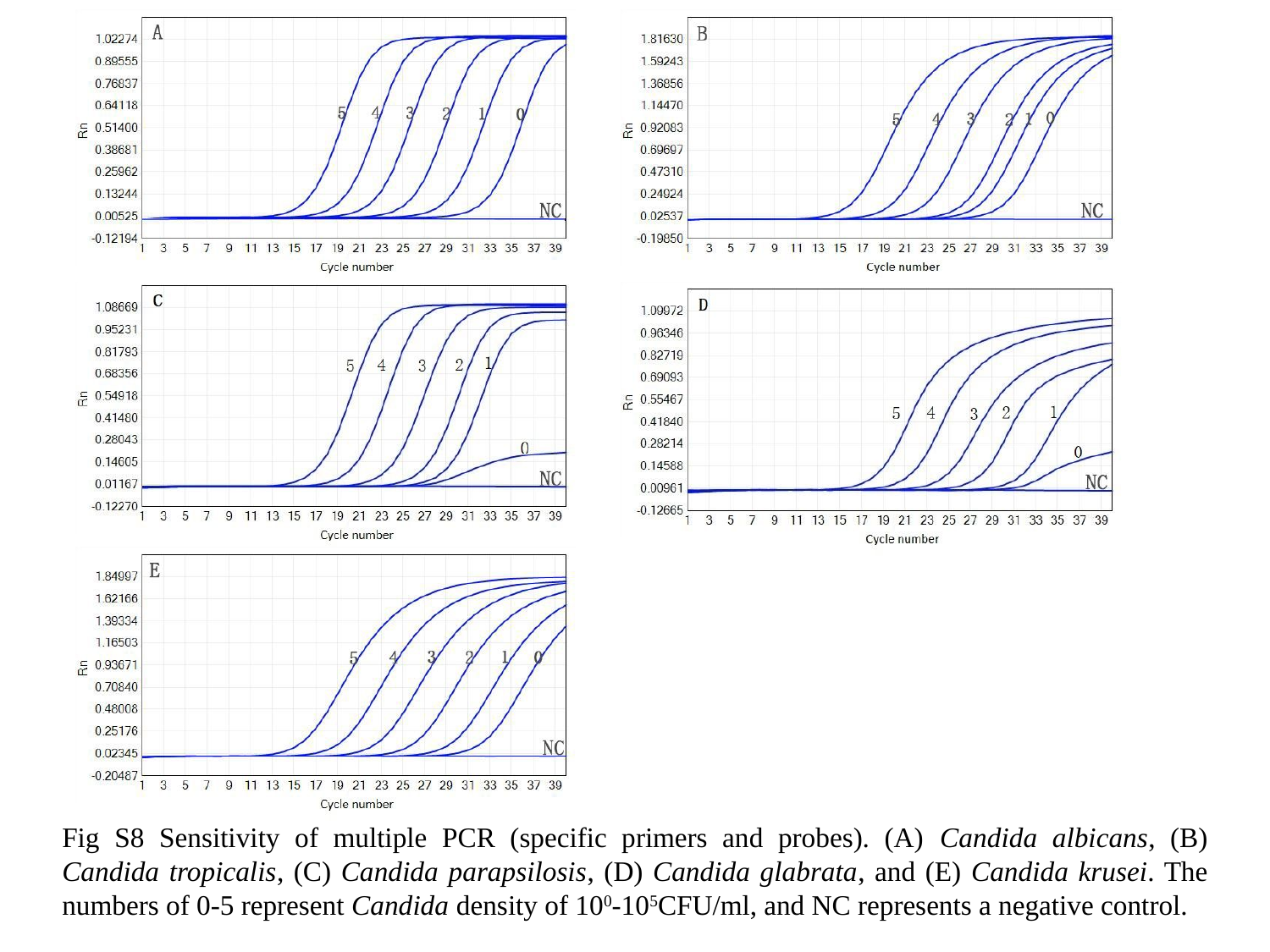

Fig S8 Sensitivity of multiple PCR (specific primers and probes). (A) Candida albicans, (B) Candida tropicalis, (C) Candida parapsilosis, (D) Candida glabrata, and (E) Candida krusei. The numbers of 0-5 represent Candida density of 100-105CFU/ml, and NC represents a negative control.

## Slide 13
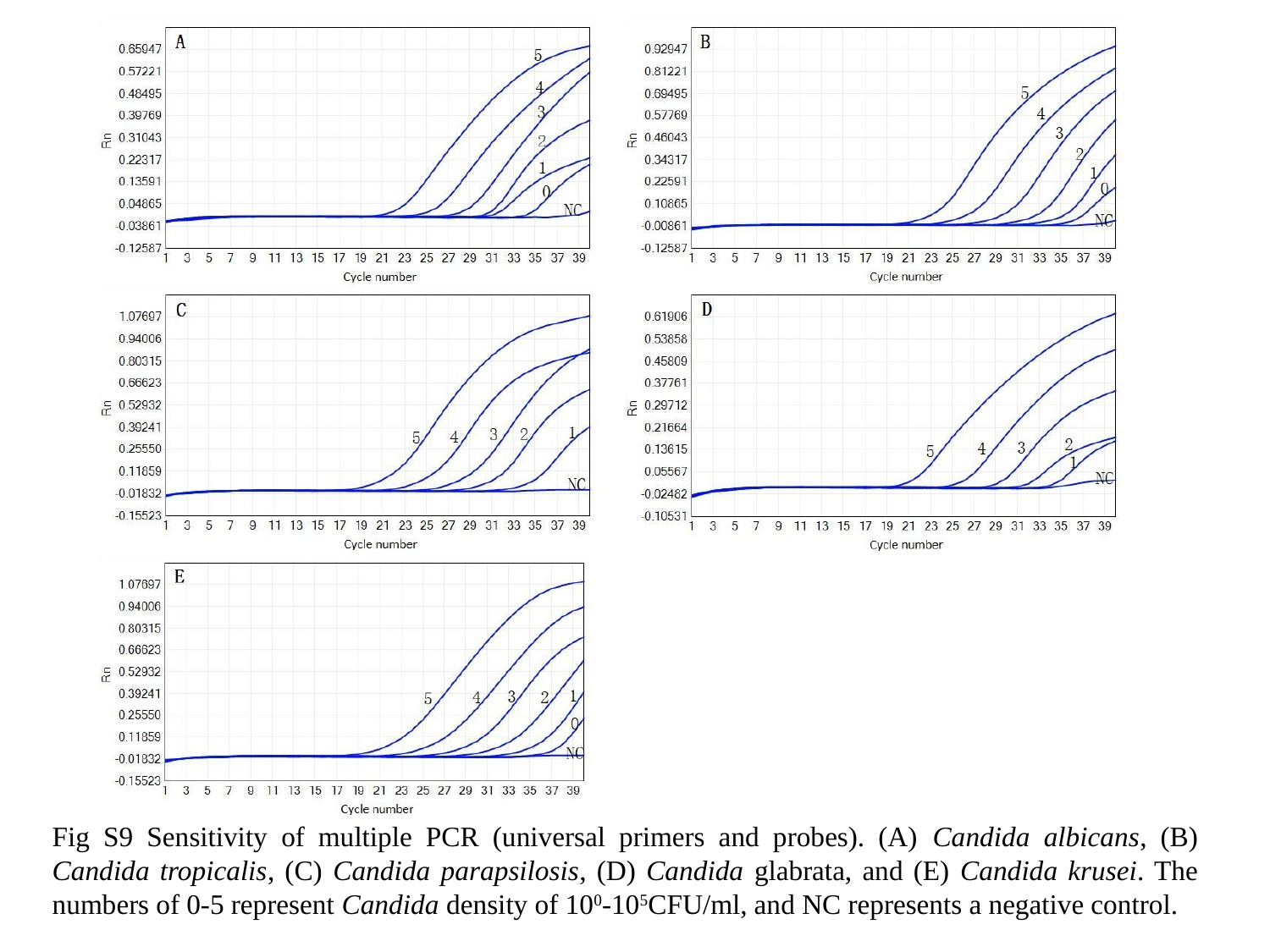

Fig S9 Sensitivity of multiple PCR (universal primers and probes). (A) Candida albicans, (B) Candida tropicalis, (C) Candida parapsilosis, (D) Candida glabrata, and (E) Candida krusei. The numbers of 0-5 represent Candida density of 100-105CFU/ml, and NC represents a negative control.

## Slide 14
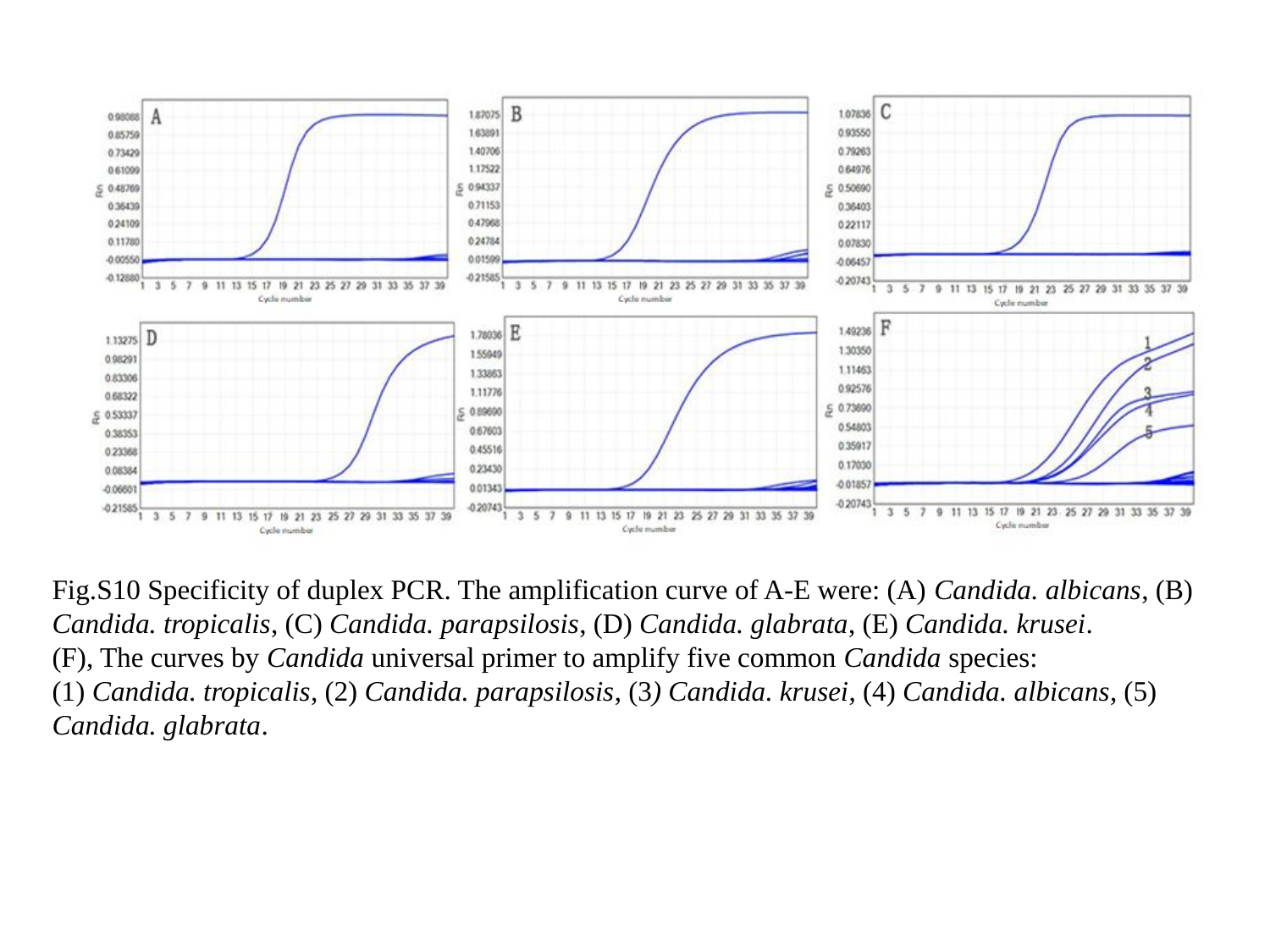

Fig.S10 Specificity of duplex PCR. The amplification curve of A-E were: (A) Candida. albicans, (B) Candida. tropicalis, (C) Candida. parapsilosis, (D) Candida. glabrata, (E) Candida. krusei.
(F), The curves by Candida universal primer to amplify five common Candida species:
(1) Candida. tropicalis, (2) Candida. parapsilosis, (3) Candida. krusei, (4) Candida. albicans, (5) Candida. glabrata.
